# The Family Physician

**Published:** 1915-09

**Authors:** T. D. Crothers

**Affiliations:** Hartford, Conn.


### EDITORIAL DEPARTMENT.

MRS. F. E. DANIEL, Publisher and Managing Editor.
DR. R. H. L. BIBB, Associate Editor.

CONTRIBUTING EDITORS:

Dr. H. Sheridan Baketel, New York, editor *Medical Times;* Dr. M. H. Boerner, Austin, Texas; Dr. G. Henri Bogart, Paris, Ill.; Dr. M. M. Carrick, Dallas, Texas; Dr. Edward H. Carey, Dean Medical Department, Baylor University, Dallas, Texas; Dr. W. L. Crosthwaite, Waco, Texas; Dr. T. D. Crothers, Hartford, Conn.; Dr. H. W. Cummings, Hearne, Texas; Dr. Oscar Dowling, New Orleans, President Louisiana State Board of Health; Havelock Ellis, M. D., London, Eng.; Dr. May Agnes Hopkins, Dallas, Texas; Dr. A. W. Fly, Galveston, Texas; Dr. G. B. Foscue, Waco, Texas; Dr. E. S. Goodhue, Holualoa, Kona, Hawaii; Dr. M. B. Grace, Seguin, Texas; Dr. Joe Gilbert, Austin, Texas; Dr. Charles H. Hughes, St. Louis, editor *Alienist and Neurologist*; Dr. James G. Kiernan, Chicago; Dr. George H. Lee, Galveston, Texas; Dr. John T. Moore, Houston, Texas; Dr. Frank Paschal, San Antonio, Texas; Dr. John Preston, Austin, Texas, Superintendent State Lunatic Asylum; Dr. G. Wilse Robinson, Kansas City, Mo.; Dr. Bacon Saunders, Fort Worth, Texas; Dr. Allen J. Smith, Philadelphia, Medical Department, University of Pennsylvania; Dr. Z. T. Scott, Austin, Texas; Dr. Ralph Steiner, Austin, Texas, President Texas State Board of Health; Dr. Walter S. Sutton, Kansas City, Mo.; Dr. A. E. Sweatland, Nacogdoches, Texas; Dr. John S. Turner, Dallas, Texas; Dr. Charles Venable, San Antonio, Texas.

### The Family Physician.

BY T. D. CROTHERS, M. D., HARTFORD, CONN.

Long ago the family physician was a distinct factor in every community. He knew all his patrons personally, and he knew their hereditary histories, occupation and what they were thinking about, and was literally able to give the most valuable services possible.

Then came the era of quacks and charlatans, new schools of medicine and finally specialists, and the old physician has disappeared. Now, the medical man decides that the case is a surgical one, or that the trouble is in the stomach or the nerves, and that he must counsel and must call in someone who is giving special attention to these particular organs, but somehow the change does not seem to work out well. There is more confusion and more doubt, and the patient gets discouraged, and goes to the drug store to buy medicines that promise to cure all the various ills the flesh is heir to.

The physician is only called in in emergencies and even then he is conscious that his services will be dispensed with any time, and on almost any pretext in favor of a specialist, or someone who is supposed to know more than the average.

The pendulum is swinging back again; in business centers and among business men the common every-day experience of retaining lawyers to draw up contracts and look over all their legal relations with other firms and prevent the possible loss that may come from mistakes, and keep them out of legal trouble, is found to be a most economical and practical measure.

The same idea is being applied in regard to a physician. To engage the services of one man who can be called on at any time and who will take pains to become acquainted with the family history and the possibility of becoming sick, is found to be equally as practical as keeping a lawyer under pay to protect his business interests.

It is the old family physician coming back again to his own, and means an accuracy in practice and certainty of results that represent scientific medicine in its highest aspects.

In the Eastern States, many families move from time to time to other localities, and they frequently inquire as to what physician they will employ, showing that they have acquired the habit of following the advice of some one man, rather than that of a number. Formerly, families called for physicians from a certain school. This is passing away. The regularly educated physician avoids being classed as practicing certain particular systems of medicine or belonging to certain schools. They justly claim the right of using any means and measures which they think will produce the best results, and when a patron is particularly anxious to have drugs from a certain school given him, they acquiesce and assert that they will treat him by the system he wishes, and in this way allay his suspicion and command his respect.

There is nothing unethical in this, or anything that reflects on the judgment of the physician. It is simply his relation to the patient. If the family believes that a certain system of drugs or a certain system of manipulation is more powerful, the physician should be ready to give it, and in this way he can break up the delusions more readily than by refusing to gratify their peculiar whims.

The old family physician never hesitated a moment to give herbs, or water or any possible drugs that the patient thought he needed. He simply assumed that they trusted to him and that his judgment would be taken as the right thing to do.

If physicians would act on this broad plain and declare that they were familiar with the various systems which are sometimes extolled in different neighborhoods, schools of medicine would disappear.

Every advance of science shows that there is only one school. Systems and creeds may vary with the man, but they represent nothing more than the opinions of the persons. All this would disappear when the idea of a family counsellor and adviser is thoroughly understood.

Then, if the physician has a large amount of common sense, he can suit his remedies to the person, and still retain the respect and confidence which will enable him to do the best kind of work along lines of preventive medicine.

Physicians, personally, should encourage the sentiment that each family and individual select a medical adviser and keep in constant touch with him in all the various changes and possibilities growing out of everyday life. There is a psychology in this field that is neglected.

A New York physician once said: "I agreed for $500 a retainer's fee, for a year, to visit a family once or twice a week, and attend them in all disabilities and illness.

"This was the beginning of a new practice which has been eminently satisfactory to me, because I have been able to evert many diseases in the first stage and make the family acquainted with means and measures of prevention. This has been followed by a number of new patrons on the same basis, and I now have a dozen or more families who are dependent on me for all questions of health, and I am able to give them most satisfactory assistance.

"If surgery is required, I take care of the cases, employ the surgeon and see that the results are satisfactory. Of course, this is an extra. If very obscure disorders occur, I call in counsel, and do not leave it to the family, and they are satisfied with my judgment."

This is the newer psychology of practical medicine, and will eventually come into everyday practice. The younger physicians should be prepared for this work, for it calls for a larger knowledge of science, hygiene, practical medicine and sociological instincts, not only to discern diseases, but to determine the best means for their cure and prevention.

There is not a village or hamlet in the country in which a family physician of this class would not find a most valuable practice. This would thoroughly drive out the quacks who promise so much, and who evidently have but one motive, and that a commercial one.

The old family doctor became really a partner with each one of his patrons, and advised them on all matters outside of medicine, and in many ways entered into partnership with them in efforts that resulted in great gain, the same as the retained lawyer in large firms; their services are so valuable that they become silent partners, and participate in the successes of the concern.

Now, a family physician would really be a silent partner in every family and his services would be appreciated and paid for on a far larger scale than any formal arrangement of so much a visit.

Of course, everything would depend on the doctor, and his wisdom in securing the family from sickness and losses.

Modern medicine makes it clear that medical assistance and help cannot be dispensed in a few minutes' calls here and there, on people who are more or less unknown to the physician. The patient must be studied at frequent intervals and the physician must know the patient, not only the physical but his mental condition, and treat both to be successful.

